# Identification of Characteristic Macromolecules of *Escherichia coli* Genotypes by Atomic Force Microscope Nanoscale Mechanical Mapping

**DOI:** 10.1186/s11671-018-2452-2

**Published:** 2018-02-02

**Authors:** Alice Chinghsuan Chang, Bernard Haochih Liu

**Affiliations:** Department of Materials Science and Engineering, National Cheng Kung University, 1 University Road, Tainan City, 701 Taiwan

**Keywords:** *E. coli* genotypes, Surface macromolecules, Atomic force microscopy, Mechanical mapping

## Abstract

The categorization of microbial strains is conventionally based on the molecular method, and seldom are the morphological characteristics in the bacterial strains studied. In this research, we revealed the macromolecular structures of the bacterial surface via AFM mechanical mapping, whose resolution was not only determined by the nanoscale tip size but also the mechanical properties of the specimen. This technique enabled the nanoscale study of membranous structures of microbial strains with simple specimen preparation and flexible working environments, which overcame the multiple restrictions in electron microscopy and label-enable biochemical analytical methods. The characteristic macromolecules located among cellular surface were considered as surface layer proteins and were found to be specific to the *Escherichia coli* genotypes, from which the averaged molecular sizes were characterized with diameters ranging from 38 to 66 nm, and the molecular shapes were kidney-like or round. In conclusion, the surface macromolecular structures have unique characteristics that link to the *E. coli* genotype, which suggests that the genomic effects on cellular morphologies can be rapidly identified using AFM mechanical mapping.

**Electronic supplementary material:**

The online version of this article (10.1186/s11671-018-2452-2) contains supplementary material, which is available to authorized users.

**Graphical Abstract:**
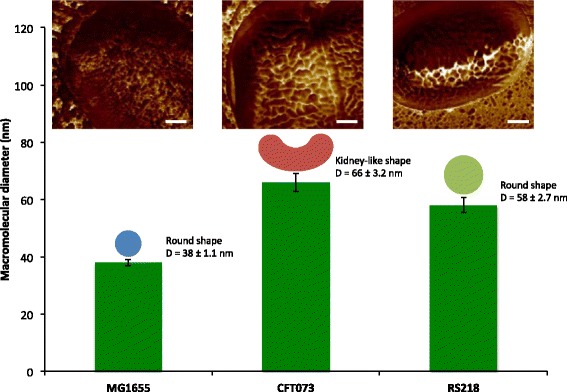
Quantification of surface macromolecules of *E. coli* cells using AFM mechanical mapping. Surface macromolecules of cellular surface of three *E. coli* genotypes, MG1655, CFT073, and RS218, were characterized with the sizes ranging from 38 to 66 nm and with round or kidney-like shapes. The topography images were colored with adhesion mapping with the scale bars = 200 nm.

## Background

Due to the inferior resolution of optical microscopy and the restricted working environment of electron microscopy, researchers in the microbial field seldom consider using the appearance of bacterial cells but instead adopt molecular or chemical analytical methods for the identification of genomic fragments, expression of proteins, etc. Not surprisingly, those methods have a number of drawbacks, including being labor-intensive and time-consuming, and thus, more straight-forward, efficient, and flexible approaches are needed. Invented in 1982 by Binnig et al., atomic force microscope (AFM) is designed to use a nanoscale probe monitored by a laser beam for the observation of a specimen’s surface with nanoscopic or even atomic resolution [1]. Imaging the morphology via a physical probe tip, this technique overcomes the resolution limit and environmental restrictions of both optical and electron microscopy and has a number of advantages, such as simple specimen preparation and flexible working environments in the ambient air or fluid conditions [2, 3]. Still, with regard to microbial studies, the current applications of AFM mainly involve qualitatively imaging the static or dynamic morphologies of bacterial cells or the expression of flagella and pili [4–6], while few studies have focused on the surface ultrastructure of microbial cells and the quantitative analysis of cellular properties.

In this research, AFM was selected for the surface study of *Escherichia coli* (*E. coli*) cells, and the shapes and dimensions of the individual bacterium were observed by the AFM topography and phase images. Furthermore, simultaneous mechanical mapping was found to reveal additional biomechanical information about the surface components, where small differences in the adhesive characteristics between the macromolecules and surrounding matrix could be detected during each physical contact between the tip and the sample. Applying such advanced techniques to the microbial fields, we have examined three *E. coli* genotypes that contained one laboratory strain and two human pathogenic strains for the identification of surface macromolecules. The results showed that the technique could offer an image resolution exceeding the scale of the AFM tip by sensing the mechanical distribution of the specimen. In conclusion, we suggest that such a development in surface science would not only provide researchers working in microbial fields with details of the cellular appearance but also contribute to our knowledge of the surface characteristics of another bio- or nanomaterial systems.

## Methods

### Microbial Samples

The three *E. coli* strains tested in this research were clinically isolated and provided by Prof. Ching-Hao Teng’s laboratory at the Institute of Molecular Medicine, National Cheng Kung University. MG1655 is the intestinal and the wild-type laboratory strain of *E. coli* K-12, and the two other strains are human pathogens—CFT073, the main cause of urinary tract infections, and RS218, associated with neonatal meningitis among infants [7].

### Functionalized Substrate

Two steps of surface modifications were applied for the covalent binding between the solid surface and microbial cells. First, 3-aminopropyltriethoxysilane (APTES, Sigma-Aldrich Co. LLC, USA) solution was used to form an initial NH_2_-functionalized layer on the surface, where the stable APTES coating was contributed to the stable Si-O bonds—Si provided by APTES and O from the oxidized surface. Possessing two COOH functions, glutaraldehyde combines to the NH_2_ function of APTES with one COOH and works with the NH_2_ on bacterial surface with the other COOH.

The clean substrates were immersed in APTES solution, with a mixture of 5% APTES in ethanol, for an hour and rinsed by ethanol and ddH_2_O. The slides were dried using nitrogen stream and then placed in glutaraldehyde solution, 2% in PBS, for overnight, and washed by PBS.

### Sample Preparation

Single colonies of the *E. coli* strains were selected from the lysogeny broth (LB) agar plates and incubated in LB broth. After the cultivation times of 12 h, the bacterial solution was then diluted 1:100 in fresh pre-equilibrated LB broth. After another 12 h for microbial cultivation, the bacterial solution was subjected to centrifuging at 1500×*g* (4000 rpm) for 3 min and resuspended in LB broth, with this process repeated twice. Two hundred microliters of the bacterial solution was dropped onto the functionalized substrate and left to rest for 30 min. The specimen was then submerged in distilled water twice to remove the unattached cells and immediately imaged under AFM in ambient air.

### AFM Characterization

An AFM instrument (Bruker Nano, Santa Barbara, CA, USA) and a silicon nitride probe with the calibrated spring constant of 0.7 N/m and tip radius of 10 nm were selected for the surface examination of microbial specimens. The AFM scan rate and line pixels were 0.5 Hz and 256 lines, respectively, for the scan size of 10 μm for the first detection of topography, and the parameters were then set as 0.3 Hz and 512 lines for the scan size of 2 μm for the detailed observation. The PeakForce quantitative nanomechanical (QNM) mode was used for the nanomechanical mapping, where the adhesive properties of the surface were calculated from the maximum attractive force among the withdrawal force-distance curves.

Our previous study on *Streptococcus mutans* showed the mechanical evolution on bacterial surface for 2 h, monitored by continuous AFM mechanical mapping, and the microbial samples were verified to remain alive within such duration [8–10]. To ensure the viability of the *E. coli* samples used in this work, the pre-studies on the bacterial samples were conducted, and the continuous change in surface adhesion for 4 h implied the cells remains alive for at least 4 h after sample preparation (shown in Additional file 1). Consequently, the microbial samples tested in this work were measured by AFM within 2 h after specimen preparation. All the *E. coli* genotypes were cultivated individually and at different times, and the AFM measurements were conducted immediately after specimen preparation. In other words, the bacterial samples were not lined up waiting for the examination, so the effects of holding time on the differences between *E. coli* strains were minimized. The quantitative data for each *E. coli* strain were collected from the measurements taken within 2 h in this study.

### Statistical Analysis

Prism (GraphPad Software, USA) was used for the statistical analysis in this work. Cellular lengths and macromolecular sizes were presented as mean values along with the standard error of the mean (SEM). The multiple comparisons between *E. coli* genotypes were processed using ordinary one-way analysis of variance (ANOVA). The confidence level of 95% (*p* < 0.05) was selected, and the asterisks indicate the degree of significant difference that was found. Sample number n of each strain was > 40.

## Results and Discussion

### Surface Ultrastructure by Multiple Mapping

**Fig. 1:**
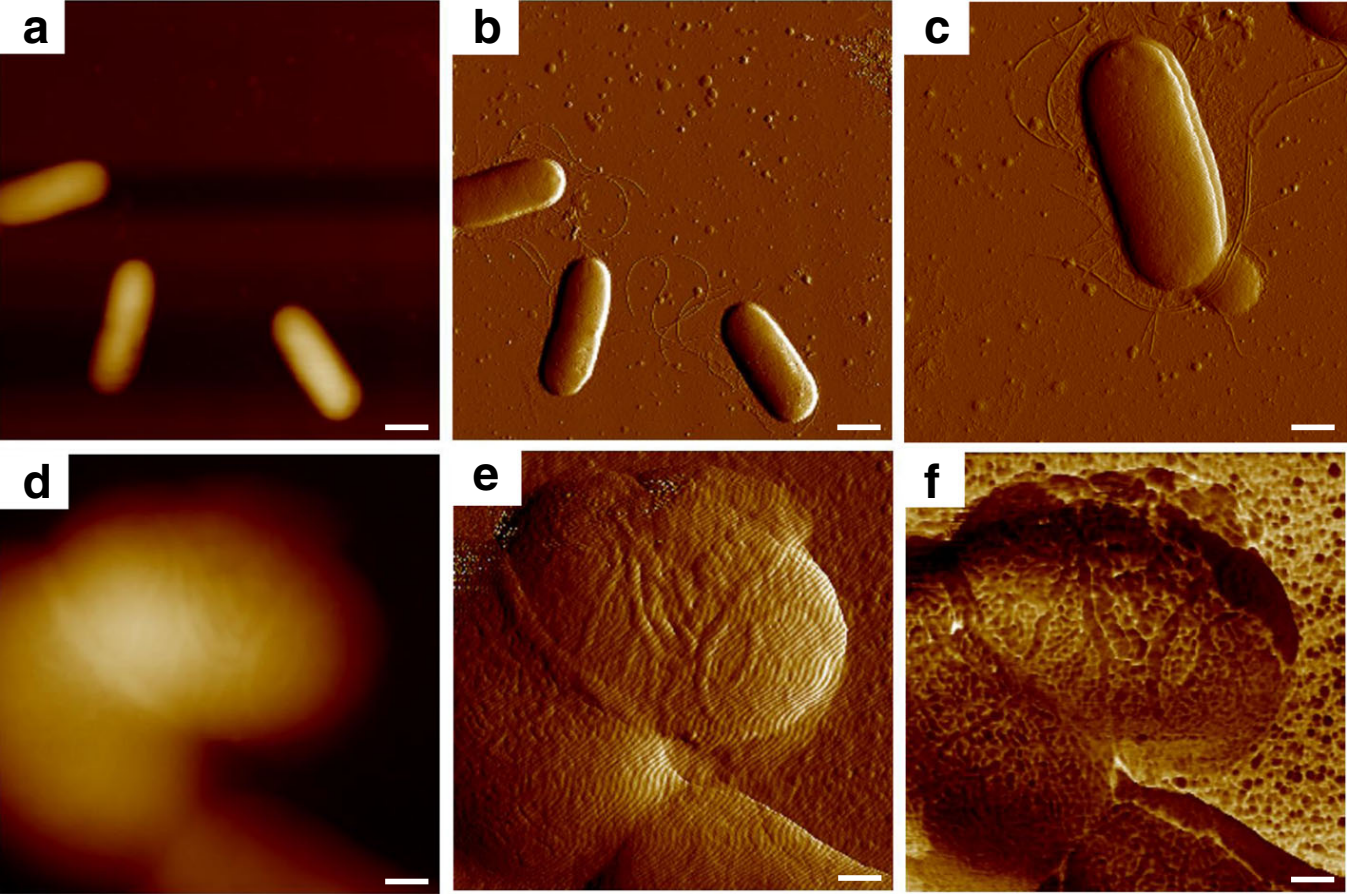
Surface ultrastructure of *E. coli* MG1655 using AFM multiple mapping. **a** and **b** were the topographical and deflection error images, and **c** was the detailed deflection error image of the bacterial cell with the expression of flagella and pili. A single cell was then focused, where **d** and **e** were the topographical and deflection error images, and **f** was the corresponding adhesion mapping. The scale bars = 1 μm in **a** and **b**, 500 nm in **c**, and 200 nm in **d**–**f**

When scanning with AFM at an observation scale of 10 μm on a bacterial sample, several single *E. coli* MG1655 cells could be seen, and the cellular shape in three dimensions could be observed from the topographic image (Fig. 1a); the clear outlines of the cells shown in two dimensions were obtained by the deflection error image (Fig. 1b), and several tubules besides the bacterial cells could be found. The wave-like filaments (Fig. 1c) were consistent with the appearance of microbial flagella reported in another work, which confirmed such finding as flagella, and the shorter and hair-like pili could also be seen [11]. When decreasing the observing area of the probe for the detailed study of single microbial cells, the topography showed a slight difference in the vertical direction among cellular surfaces, as shown in Fig. 1d, and the deflection error image seemed to provide more morphological information while the collected environmental noise was too much to investigate the ultrastructure of the cellular surface (Fig. 1e). When the simultaneous biomechanical properties of the specimen were measured during the contact between tip and tested object, it was found that the bacterial surface was actually composed of a huge amount of macromolecules with specific shape and size, as was revealed by the adhesive force mapping (Fig. 1f); thus, the morphological resolution in topographic and deflection error images were further improved with biomechanical information.

**Fig. 2:**
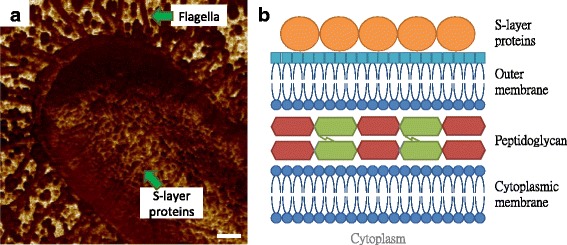
Illustration of vertical and surface structures of *E. coli* cells. **a** The surface macromolecules on *E. coli* MG1655 cell imaged by AFM adhesion mapping. **b** The molecular architecture of cell envelope in Gram-negative microbes, which consists of cytoplasmic membrane, peptidoglycan, outer membrane, and S-layer. The scale bar = 200 nm

In Fig. 2a, the expression of flagella by *E. coli* MG1655 could be evidently seen, where the size of filaments was similar with those in Fig. 1b, with the adhesion properties relatively lower than the substrate. In addition, the bacterial surface was found to be composed of circular components that were characterized to be less adhesive when compared to the surrounding matrix. This observation is similar to our previous findings on the tissue layers of mice skin, and the recurring and comparable grains were considered to be macromolecules, whose structure is more dense and consistent than those seen in the intermolecular region so that the differences in adhesion performance could be easily sensed [12]. The outermost layer of the cellular envelope in Gram-negative bacteria is a layer of self-assembly proteins, as illustrated in Fig. 2b, which is known as a surface layer (S-layer) protein [13]. S-layer structures were traditionally measured by electron microscopy while the requirements of vacuum environments and conductive coating lost the native and real-time information about the proteins. Although some researches extracted the S-layer proteins and reassembled them onto mica substrate for the AFM scanning, the results lacked for the in situ and real-time performance of the S-layer structure [14, 15]. Based on the cellular architecture and some previous images of microbial surface by electron microscopy, we considered the observed macromolecules as S-layer proteins [16].

Comparing the AFM and transmission electron microscopy (TEM) measurements, the former has several advantages over the latter, such as simpler specimen preparation, less restricted experimental requirements, and more biological-friendly imaging applications. The selection of TEM in the biological field is generally selected due to its ability to see the intercellular organelles and the ultra-resolution (typically nanometer or sub-nanometer) imaging that can be obtained. The AFM adhesion mapping in the current work improved the feature resolution and presented the arrangements of surface macromolecules in a way that did not depend on the tip size, but instead on the intrinsic structure of the sample itself. Furthermore, this approach enables nanoscale resolution of microbial surface throughout a range of tens of micrometer square area.

### Genomic-Manipulating Differences in Morphological Characteristics

**Fig. 3:**
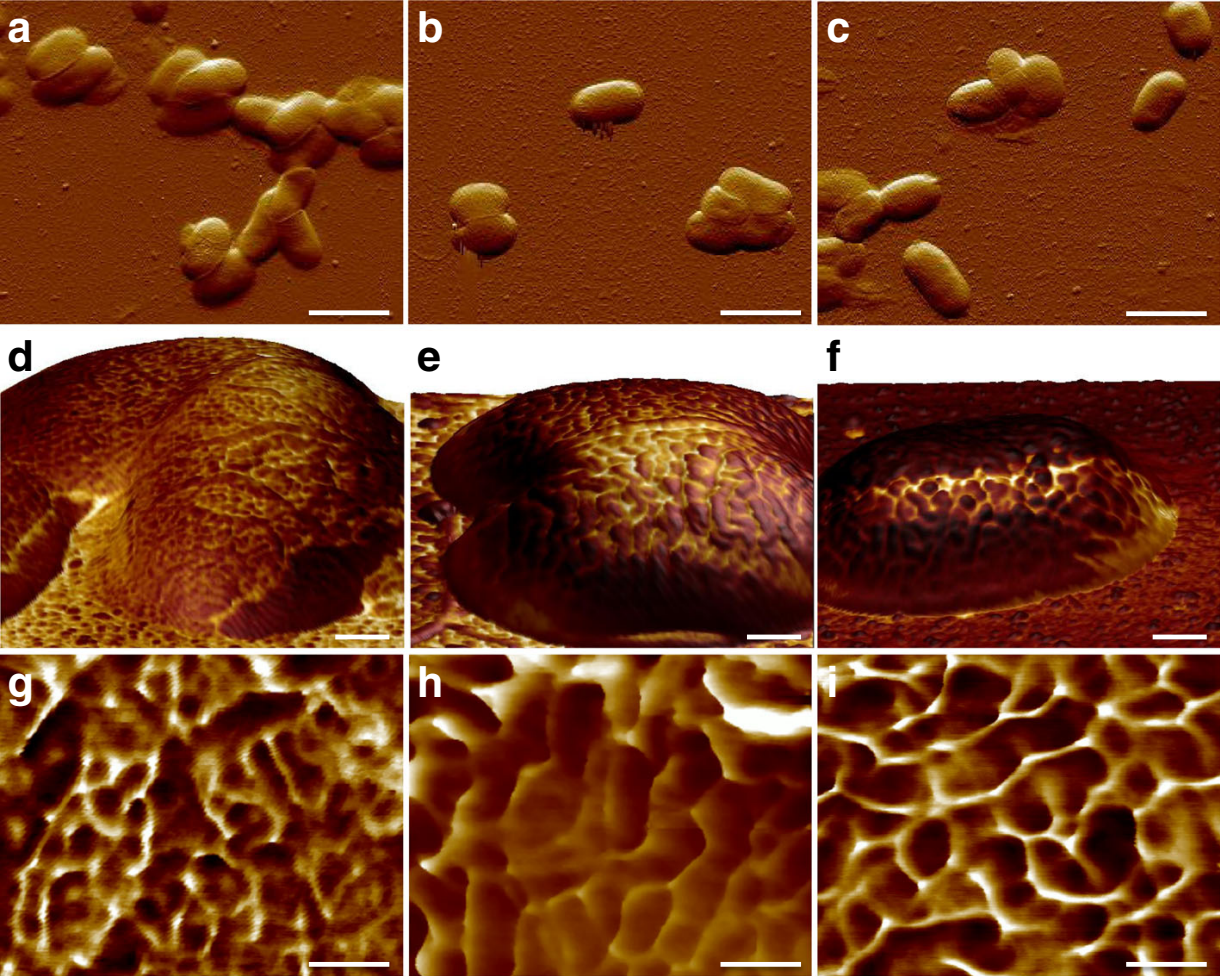
Morphological characteristics of the *E. coli* genotypes. The upper row displayed the deflection error images of **a** MG1655, **b** CFT073, and **c** RS218. The middle row showed the 3D topographies colored with adhesion mapping on **d** MG1655, **e** CFT073, and **f** RS218 cells. The lower row was the detailed adhesion mapping on **g** MG1655, **h** CFT073, and **i** RS218. In adhesion mapping, the darker color referred to less adhesion performances and vice versa. The scale bars were 2 μm for **a**–**c**, 200 nm for **d**–**f**, and 100 nm for **g**–**i**

**Fig. 4:**
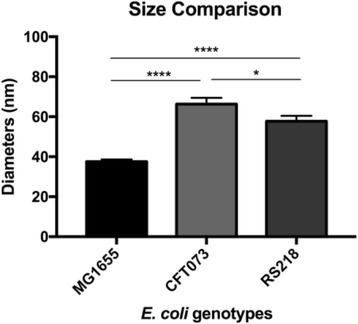
Molecular sizes of *E. coli* genotypes. The diameters of surface proteins were detected by AFM and processed via one-way ANOVA for the multiple comparisons. ****p < 0.001 and *p < 0.05

After observing the surface ultrastructure of *E. coli* MG1655 cells, one issue of interest is how the macromolecules are arranged in other strains. The human pathogens *E. coli* CFT073 and RS218 were thus examined by AFM with the same experimental parameters, and there were no significant differences in cellular shapes and dimensions between these three genotypes at the feature size of 10 μm, seen in Fig. 3a–c. Adhesive force mapping was used to identify the S-layer proteins, and dissimilar structures with various shapes and sizes were detected among the different *E. coli* strains, as shown in Fig. 3d–f. For the ease of comparison, the detailed adhesion mapping images of the *E. coli* strains were displayed in Fig. 3g–i. The surface macromolecules were characterized as having a round shape in MG1655 and RS218 cells, although different molecular diameters, which were 38 ± 1.1 nm (*n* = 80) for MG1655 and 58 ± 2.7 nm (*n* = 46) for RS218. On the other hand, the CFT073 cells possessed a unique shape of S-layer proteins, which were kidney-like with the length difference between two end points of 66 ± 3.2 nm (*n* = 44). After analyzing the sizes of the S-layer proteins of these three genotypes for multiple comparisons, the results demonstrated the significant differences between these strains (Fig. 4).

Microbial S-layers are reported to play important roles in many functions, which contain protecting cells from severe environments, attacks of phagocytosis, and predatory bacterium. In addition, S-layers also serve as adhesin that enables the effective colonization [17]. The S-layer structures have been well studied by TEM and been categorized into various lattice types with the space between center-to-center in the range of 4–35 nm [16]. The variation between our AFM results and TEM reports from the literature was thought as the different imaging methodologies, where TEM gives the 2D morphology of S-layer structure and AFM captures the 3D topography that includes the multiple influences contributed by cellular radian and roughness and the geometry of AFM probe.

Different types of S-layer structures were originally thought possible to use their various taxonomic characteristics to distinguish among bacterial species, although it was then found that even for single species, the microbial stains could possess different lattices of proteins [13, 16, 18, 19]. While some studies investigated the role of the S-layer in filament formation, the types of proteins on cell membranes and the genomic diversities in size among *E. coli* genotypes, the differences in S-layer proteins have seldom been noted [20–22]. The results of the current study revealed the differences in morphological characteristics among *E. coli* MG1655, CFT073, and RS218 and suggest that the appearances of the surface macromolecules were probably specific to individual *E. coli* genotype.

## Conclusions

In this work, genomic-specific nanostructural information about the bacterial surface was detected by AFM mechanical mapping, which distinguished the adhesive differences between the macromolecules and the surrounding matrix. The surface macromolecules of the microbial cells were considered as the surface layer proteins, according to the molecular architecture of Gram-negative microbes. The arrangements and sizes of those macromolecules were found to be specific to the tested *E. coli* genotypes with distinct shapes and sizes, with these differences shown to be significant by statistical analysis. In conclusion, we consider the bacterial S-layer structure is genome-dependent and can be the potential method for the rapid diagnosis of microbe-associated diseases or microbial strains. To implement the practical application of S-layer characteristic, the examination on more bacterial genotypes is required for the complement catalog. We are currently establishing the database connecting bacterial morphological characteristics and the physiological/pathological performances and believe that it will be a promising progress for the practical application of AFM examination.

## Additional file

Additional file 1: Figure S1. Mechanical evolution of *E. coli* cellular surface at time sequences. The images were the adhesion mapping of an *E. coli* MG1655 cell, and the continuous change in adhesion properties was monitored by AFM. The images were captured after (a) 30 min, (b) 60 min, (c) 90 min, (d) 120 min, (e) 150 min, (f) 180 min, (g) 210 min, and (h) 240 min from specimen preparation. The scale bars = 200 μm. (DOCX 1607 kb)

## Abbreviations

AFM: Atomic force microscope
ANOVA: One-way analysis of variance
APTES: 3-Aminopropyltriethoxysilane
LB: Lysogeny broth
QNM: Quantitative nanomechanical
S-layer: Surface layer
TEM: Transmission electron microscopy

## References

[1] Binnig G, Quate CF, Gerber C (1986). Atomic force microscope. Phys Rev Lett.

[2] Sahin O, Erina N (2008). High-resolution and large dynamic range nanomechanical mapping in tapping-mode atomic force microscopy. Nanotechnology.

[3] Chang AC, Liao J-D, Liu BH (2016). Practical assessment of nanoscale indentation techniques for the biomechanical properties of biological materials. Mech Mater.

[4] Su HN, Chen ZH, Song XY, Chen XL, Shi M, Zhou BC, Zhao X, Zhang YZ (2012). Antimicrobial peptide trichokonin VI-induced alterations in the morphological and nanomechanical properties of *Bacillus subtilis*. PLoS One.

[5] Huang Q, Wu H, Cai P, Fein JB, Chen W (2015). Atomic force microscopy measurements of bacterial adhesion and biofilm formation onto clay-sized particles. Sci Rep.

[6] Tripathi P, Dupres V, Beaussart A, Lebeer S, Claes IJ, Vanderleyden J, Dufrêne YF (2012). Deciphering the nanometer-scale organization and assembly of *Lactobacillus rhamnosus* GG pili using atomic force microscopy. Langmuir.

[7] Kaper JB, Nataro JP, Mobley HL (2004). Pathogenic *Escherichia coli*. Nat Rev Microbiol.

[8] Liu BH, Yu L-C (2017). In-situ, time-lapse study of extracellular polymeric substance discharge in *Streptococcus mutans* biofilm. Colloids Surf B: Biointerfaces.

[9] Liu BH, Li K-L, Huang W-K, Liao J-D (2013). Nanomechanical probing of the septum and surrounding substances on *Streptococcus mutans* cells and biofilms. Colloids Surf B: Biointerfaces.

[10] Liu BH, Li K-L, Kang K-L, Huang W-K, Liao J-D (2013). In situ biosensing of the nanomechanical property and electrochemical spectroscopy of *Streptococcus mutans*-containing biofilms. J Phys D Appl Phys.

[11] Gillis A, Dupres V, Delestrait G, Mahillon J, Dufrêne YF (2012). Nanoscale imaging of *Bacillus thuringiensis* flagella using atomic force microscopy. Nano.

[12] Chang AC, Liu BH, Shao PL, Liao JD (2017). Structure-dependent behaviours of skin layers studied by atomic force microscopy. J Microsc.

[13] Sleytr UB, Beveridge TJ (1999). Bacterial S-layers. Trends Microbiol.

[14] Gufler PC, Pum D, Sleytr UB, Schuster B (2004). Highly robust lipid membranes on crystalline S-layer supports investigated by electrochemical impedance spectroscopy. Biochim Biophys Acta.

[15] Shevchuk AI, Frolenkov GI, Sánchez D, James PS, Freedman N, Lab MJ, Jones R, Klenerman D, Korchev YE (2006). Imaging proteins in membranes of living cells by high-resolution scanning ion conductance microscopy. Angew Chem Int Ed Engl.

[16] Sleytr UB, Schuster B, Egelseer EM, Pum D (2014). S-layers: principles and applications. FEMS Microbiol Rev.

[17] Fagan RP, Fairweather NF (2014). Biogenesis and functions of bacterial S-layers. Nat Rev Microbiol.

[18] Schäffer C, Messner P (2004). Surface-layer glycoproteins: an example for the diversity of bacterial glycosylation with promising impacts on nanobiotechnology. Glycobiology.

[19] Ilk N, Egelseer EM, Sleytr UB (2011). S-layer fusion proteins—construction principles and applications. Curr Opin Biotechnol.

[20] Lederer FL, Günther TJ, Raff J, Pollmann K (2011). *E. coli* filament formation induced by heterologous S-layer expression. Bioeng Bugs.

[21] Walters MS, Mobley HL (2009). Identification of uropathogenic *Escherichia coli* surface proteins by shotgun proteomics. J Microbiol Methods.

[22] Xie Y, Kolisnychenko V, Paul-Satyaseela M, Elliott S, Parthasarathy G, Yao Y, Plunkett G, Blattner FR, Kim KS (2006). Identification and characterization of *Escherichia coli* RS218-derived islands in the pathogenesis of *E. coli* meningitis. J Infect Dis.

