# Supplementary material for: Identification of Characteristic Macromolecules of Escherichia coli Genotypes by Atomic Force Microscope Nanoscale Mechanical Mapping
Source: Nanoscale Res Lett. 2018 Feb 2;13:35. doi: 10.1186/s11671-018-2452-2 (PMC5796958; doi:10.1186/s11671-018-2452-2)
Supplement: Additional file 1: Figure S1. — Mechanical evolution of E. coli cellular surface at time sequences. The images were the adhesion mapping of an E. coli MG1655 cell, and the continuous change in adhesion properties was monitored by AFM. The images were captured after (a) 30 min, (b) 60 min, (c) 90 min, (d) 120 min, (e) 150 min, (f) 180 min, (g) 210 min, and (h) 240 min from specimen preparation. The scale bars = 200 μm. (DOCX 1607 kb) [file 11671_2018_2452_MOESM1_ESM.docx]

**Identification of characteristic macromolecules of *Escherichia coli* genotypes by AFM nanoscale mechanical mapping**

A. C. Chang and B. H. Liu*

**Additional file 1**

**Figure S1. Mechanical evolution of *E. coli* cellular surface at time sequences.** The images were the adhesion mapping of an *E. coli* MG1655 cell and the continuous change in adhesion properties were monitored by AFM. The images were captured after (a) 30 minutes, (b) 60 minutes, (c) 90 minutes, (d) 120 minutes, (e) 150 minutes, (f) 180 minutes, (g) 210 minutes, and (h) 240 minutes from specimen preparation. The scale bars = 200 µm.
